# Towards active lower limb prosthetic systems: design issues and solutions

**DOI:** 10.1186/s12938-016-0283-x

**Published:** 2016-12-19

**Authors:** Oliver Christ, Philipp Beckerle

**Affiliations:** 1 School of Applied Psychology, Institute Humans in Complex Systems, University of Applied Sciences and Arts Northwestern Switzerland, Olten, Switzerland; 2 Institute for Mechatronic Systems in Mechanical Engineering, Technische Universität Darmstadt, Darmstadt, Germany

**Keywords:** Prosthetics, Lower limb amputation, Engineering design, Biomechanics, Psychological adjustment

## Background

A prosthesis is a crucial technical substitute that should restore biomechanical function and body integrity for people with lower limb loss or congenital limb absence [1]. Within the last decades, lower limb prostheses developed from passive mechanisms to adaptive mechatronic systems [2]. Contemporary, such prostheses evolve to robotic systems providing powered locomotion support by drives as shown in [3, 4]. According to the review in from [5], 21 different active lower limb prostheses are found in the research literature. With such technologies, various new research questions arise and the idea of prosthesis technology simulation is being discussed [6, 7].

Technically, the mechatronic design of actuators and kinematics as well as the development of suitable control algorithms are challenging tasks [3, 4]. A promising approach to actuation is found in compliant actuators and kinematics that store and transfer energy between gait phases [8]. To command those actuators, controllers that mimic biological function during different gait situations, speeds, and transitions as the one propose by Grimmer et al. [9] are required.

Analyzing human biomechanics with and without considering the prosthetic system is a crucial basis for design and control that provides requirements and constrains [10]. Further, biomechanical studies can be used to assess the utility of active prostheses and indicate that those improve amputee gait [3, 11].

As prostheses are not only used by people, but aim at replacing lost parts of amputees’ bodies, human factors show significant impact on prosthetic development from a psychological perspective [12–14]. Those comprise aspects such as acceptance [15] and integration to the body schema [16–20].

Those human factors impact technical design [21, 22] and need psychological methods to be surveyed [23–25] and considered in design [26]. Additionally, insights regarding human factors can be used to develop and improve novel techniques for movement rehabilitation, e.g., gait training in virtual reality environments [27–29].

The articles in this supplement contribute to those topics by tackling elastic actuation, gait recognition and control, biomechanical analysis and simulation, human factors, and virtual reality rehabilitation.
